# Angioplasty for pulmonary vein stenosis after atrial fibrillation ablation

**DOI:** 10.1093/europace/euag164

**Published:** 2026-07-01

**Authors:** André Fitzner, Martin Martinek, Helmut Pürerfellner, Georgios Kollias, Michael Derndorfer, Christine Lemeš, Thomas Sturmberger

**Affiliations:** Department of Cardiology, Angiology & Intensive Care Medicine, Ordensklinikum Linz Elisabethinen, Fadingerstr.1, Linz 4020, Austria; Department of Cardiology, Angiology & Intensive Care Medicine, Ordensklinikum Linz Elisabethinen, Fadingerstr.1, Linz 4020, Austria; Department of Cardiology, Angiology & Intensive Care Medicine, Ordensklinikum Linz Elisabethinen, Fadingerstr.1, Linz 4020, Austria; Department of Cardiology, Angiology & Intensive Care Medicine, Ordensklinikum Linz Elisabethinen, Fadingerstr.1, Linz 4020, Austria; Department of Cardiology, Angiology & Intensive Care Medicine, Ordensklinikum Linz Elisabethinen, Fadingerstr.1, Linz 4020, Austria; Department of Cardiology, Angiology & Intensive Care Medicine, Ordensklinikum Linz Elisabethinen, Fadingerstr.1, Linz 4020, Austria; Department of Cardiology, Angiology & Intensive Care Medicine, Ordensklinikum Linz Elisabethinen, Fadingerstr.1, Linz 4020, Austria

**Keywords:** Pulmonary vein stenosis, Atrial fibrillation ablation, Pulmonary vein stenting, Restenosis, Patient adherence/compliance, Stent patency

## Abstract

**Graphical Abstract:**
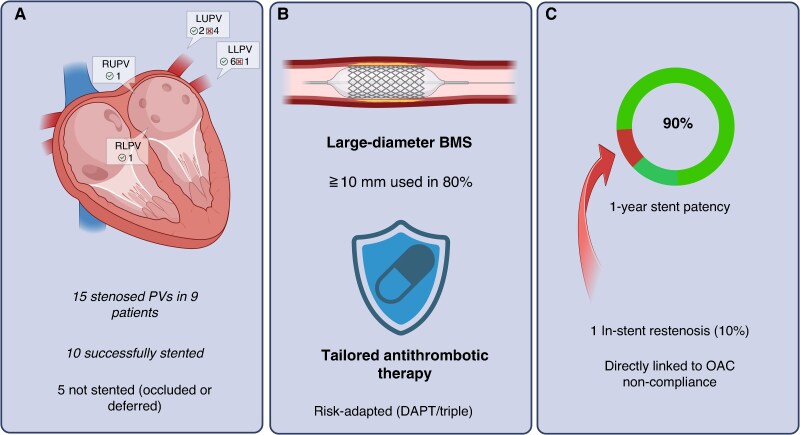
Interventional management of pulmonary vein stenosis. (*A*) Anatomical distribution of stenotic pulmonary veins (*n* = 15) and per-vein procedural success rates. The labels indicate successfully stented (check mark) vs. deferred or occluded (cross) veins for each anatomical location. (*B*) Critical procedural factors for long-term success: the use of large-diameter stents (≥10 mm), which were successfully implanted in 80% of treated veins, and a tailored, risk-adapted antithrombotic therapy. (*C*) Long-term outcome demonstrating 90% stent patency at 1 year. The single case of restenosis (10%) was directly linked to documented medication non-adherence. BMS, bare metal stent; DAPT, dual antiplatelet therapy; LLPV, left lower pulmonary vein; LUPV, left upper pulmonary vein; OAC, oral anticoagulation; RLPV, right lower pulmonary vein; RUPV, right upper pulmonary vein; Created in BioRender. Fitzner, A. (2026) https://BioRender.com/hlhysjl.

Interventional management of pulmonary vein stenosis. (*A*) Anatomical distribution of stenotic pulmonary veins (*n* = 15) and per-vein procedural success rates. The labels indicate successfully stented (check mark) vs. deferred or occluded (cross) veins for each anatomical location. (*B*) Critical procedural factors for long-term success: the use of large-diameter stents (≥10 mm), which were successfully implanted in 80% of treated veins, and a tailored, risk-adapted antithrombotic therapy. (*C*) Long-term outcome demonstrating 90% stent patency at 1 year. The single case of restenosis (10%) was directly linked to documented medication non-adherence. BMS, bare metal stent; DAPT, dual antiplatelet therapy; LLPV, left lower pulmonary vein; LUPV, left upper pulmonary vein; OAC, oral anticoagulation; RLPV, right lower pulmonary vein; RUPV, right upper pulmonary vein; Created in BioRender. Fitzner, A. (2026) https://BioRender.com/hlhysjl.

## Introduction

Pulmonary vein stenosis (PVS) remains a rare but challenging complication following atrial fibrillation (AF) ablation, associated with significant morbidity.^1,2^ While recent expert consensus statements provide detailed guidance on ablation techniques to minimize collateral damage,^3^ data regarding the optimal interventional treatment of established PVS remain limited.^4,5^

Current management strategies vary widely, particularly regarding stent selection and post-interventional antithrombotic therapy. As recently highlighted by Tokuda et al.^6^ and demonstrated by Suntharos et al.^7^ restenosis rates reported in the literature historically range up to 25%, often attributed to elastic recoil or neointimal hyperplasia.

In this study, we report the long-term outcomes of a specific interventional strategy utilizing large-diameter stents (≥10 mm) combined with an individualized antithrombotic regimen. We aimed to determine the incidence of restenosis and identify factors contributing to sustained stent patency in a real-world cohort.

## Methods

This retrospective single-centre study included all patients presenting with symptomatic PVS requiring intervention at the Ordensklinikum Linz between October 2021 and December 2023. Data were retrieved from the hospital information system and the national electronic health record (Elektronische Gesundheitsakte, ELGA). The study was approved by the Ethics Committee of the Medical Faculty of the JKU (No. 1070/2024).

### Interventional procedure

Procedural planning included CT angiography to assess PV anatomy. Access was obtained via transseptal puncture. A 3D electroanatomical map (CARTO 3, Biosense Webster) was created to reconstruct left atrial geometry and localize PV ostia, facilitating catheter engagement even in subtotal occlusions. Stenoses were pre-dilated with balloon catheters followed by the implantation of large-diameter bare metal stents (BMS), aiming for a stent diameter of ≥10 mm to maximize acute luminal gain and prevent elastic recoil.

### Antithrombotic regimen

Post-interventional antithrombotic therapy followed a risk-adapted algorithm:

1. **Standard:** Dual antiplatelet therapy (DAPT: aspirin + clopidogrel) for 3–6 months, followed by oral anticoagulation (OAC) monotherapy.
2. **High-risk (thrombus/slow flow):** Escalation to triple therapy (OAC + DAPT) for 1–3 months.
3. **High bleeding risk / specific indications:** Combination of single antiplatelet therapy (SAPT) and a direct oral anticoagulant (DOAC).

### Follow-up and endpoints

Follow-up included clinical assessment and CT angiography at 90 days and 12 months. The primary endpoint was stent patency. Significant restenosis was defined as >50% luminal narrowing or complete occlusion.

## Results

A total of nine patients (mean age 55.8 ± 11 years, 8 male) with 15 stenotic PVs were treated. All patients had undergone prior AF ablation (mean 1.4 ± 1.7 procedures). Due to our institution's role as a tertiary referral centre receiving external PVS cases, establishing a true incidence rate relative to our total pulmonary vein isolation volume was methodologically not feasible.

### Procedural outcomes

The left-sided pulmonary veins were affected more frequently (*n* = 13) than the right-sided veins (*n* = 2). Overall, intervention was successful in 10 of 15 stenotic veins (67%). Specifically, stenting was successful in six of seven left inferior pulmonary veins (85.7%) and two of six left superior pulmonary veins (33.3%), while both right-sided stenoses were successfully treated. Among the remaining five veins, stenting was either anatomically not feasible due to complete occlusion, or the intervention was deferred to limit prolonged procedure time and ensure patient safety. In 80% of treated veins, a 10×19 mm stent was successfully implanted.

### Antithrombotic therapy and follow-up

At discharge, five patients received standard DAPT, three patients were prescribed triple therapy due to high thrombotic burden, and one patient was discharged on a combination of SAPT and a DOAC due to individual bleeding risk assessment. After a mean follow-up of 363 days, stent patency was confirmed in 90% of treated veins. Only one patient developed significant restenosis (complete occlusion), which occurred in the context of documented non-adherence to the prescribed antithrombotic therapy.

## Discussion

This study demonstrates that angioplasty with large-diameter stents yields excellent long-term outcomes in patients with PVS.^8^ Our 10% restenosis rate aligns with recent data from Suntharos et al.^7^ and compares favourably to meta-analyses reporting rates up to 25%.^9,10^

While drug-eluting stents are often discussed, they are currently not commercially available in the large diameters (≥10 mm) required for PV stenting. Therefore, we prioritized large-diameter BMS, which also provide superior radial strength to resist elastic recoil, a dominant mechanism in PVS.

A critical finding is the importance of strict adherence to antithrombotic therapy. The only restenosis in our cohort was directly linked to non-compliance. Our risk-adapted regimen (escalating to triple therapy for high-risk features) appears effective in preventing early stent thrombosis.

### Limitations

The small sample size reflects the rarity of the condition. As a retrospective single-centre study, selection bias cannot be excluded.

## Conclusion

Pulmonary vein stenosis stenting with large-lumen stents is safe and effective. Optimized stent sizing and strict adherence to individualized antithrombotic therapy are key to ensuring long-term patency.

## Data Availability

The data underlying this article cannot be shared publicly due to the privacy of individuals that participated in the study. The data will not be shared.

## Notes

**Declaration of Generative AI and AI-assisted technologies in the writing process:** During the preparation of this work, the author used ChatGPT (GPT-5, OpenAI) and Gemini (Google) to assist in language refinement and translation. After using these tools, the authors reviewed and edited the content as needed and takes full responsibility for the content of the publication.
